# Translation of MMTV Gag requires nuclear events involving splicing motifs in addition to the viral Rem protein and RmRE

**DOI:** 10.1186/1742-4690-9-8

**Published:** 2012-01-25

**Authors:** Ioana Boeras, Michael Sakalian, John T West

**Affiliations:** 1 Department of Microbiology and Immunology, University of Oklahoma Health Sciences Center, Oklahoma City, OK, USA

**Keywords:** Betaretrovirus, *gag*, Rem, post-transcriptional regulation

## Abstract

**Background:**

Retroviral Gag proteins are encoded in introns and, because of this localization, they are subject to the default pathways of pre-mRNA splicing. Retroviruses regulate splicing and translation through a variety of intertwined mechanisms, including 5'- post-transcriptional control elements, 3'- constitutive transport elements, and viral protein RNA interactions that couple unspliced and singly spliced mRNAs to transport machinery. Sequences within the *gag *gene termed inhibitory or instability sequences also appear to affect viral mRNA stability and translation, and the action of these sequences can be countered by silent mutation or the presence of RNA interaction proteins like HIV-1 Rev. Here, we explored the requirements for mouse mammary tumor virus (MMTV) Gag expression using a combination of *in vivo *and *in vitro *expression systems.

**Results:**

We show that MMTV *gag *alleles are inhibited for translation despite possessing a functional open reading frame (ORF). The block to expression was post-transcriptional and targeted the mRNA but was not a function of mRNA transport or stability. Using bicistronic reporters, we show that inhibition of *gag *expression imparted a block to both cap-dependent and cap-independent translation onto the mRNA. Direct introduction of *in vitro *synthesized *gag *mRNA resulted in translation, implying a nuclear role in inhibition of expression. The inhibition of expression was overcome by intact proviral expression or by flanking *gag *with splice sites combined with a functional Rem-Rem response element (RmRE) interaction.

**Conclusions:**

Expression of MMTV Gag requires nuclear interactions involving the viral Rem protein, its cognate binding target the RmRE, and surprisingly, both a splice donor and acceptor sequence to achieve appropriate signals for translation of the mRNA in the cytoplasm.

## Background

Eukaryotic mRNAs typically contain intronic sequences that must be removed by ubiquitously acting splicing mechanisms prior to nuclear export. Splicing occurs co-transcriptionally and affects pre-mRNA stability, 5' and 3' end formation, nuclear export, cytoplasmic trafficking and stability, as well as translation [1,2]. As the pre-mRNA emerges from PolII it is complexed with a very dynamic series of proteins that forms the mRNP (messenger ribonucleoprotein). Components of the mRNP and changes to those components regulate nuclear and cytoplasmic steps in mRNA metabolism [1-4].

Post-integration, retroviruses largely utilize host transcription and translation mechanisms to proceed through the replication cycle. Retroviruses initiate a complex transcription profile that is driven by alternative splicing and results in three distinct RNA species: unspliced genome-length transcripts, singly spliced transcripts, and fully spliced transcripts. For all retroviruses, the primary structural protein Gag is encoded by an intron in the unspliced genomic RNA. Thus, Gag translation requires unique mechanisms to overcome the default eukaryotic splicing pathway and transport an intron-containing mRNP to the cytoplasm.

In parallel with the evolving complexity of their expression programs, retroviruses have acquired increasingly complex mechanisms to regulate mRNA processing and export. One of these strategies is the adoption of suboptimal splice sites and splicing enhancers and suppressors that regulate the capacity of cellular machinery to cleave the mRNA prior to export [5-8]. This strategy is adopted by the avian sarcoma leukosis viruses (ASLV), murine leukemia virus (MLV), and other simple retroviruses, but it is also maintained in more complex retroviral species. In addition to suboptimal splicing, some betaretroviruses contain intronic RNA structures, called constitutive transport elements (CTE), which directly bridge the interaction of singly-spliced and genomic mRNAs with proteins of the TAP transport pathway [9]. Complex retroviruses add an additional level of control by encoding a regulator of RNA export from a spliced viral mRNA and utilizing a different export pathway. These proteins, the prototype of which is human immunodeficiency virus type 1 (HIV-1) Rev, interact with an RNA structural element in underspliced *gag *or *env *mRNAs and couple them to the Crm1 export pathway [10-15].

MMTV has been extensively studied as a model for breast cancer. Much emphasis has been given to defining the hormonal regulation of transcription and the mechanisms of cellular transformation [16,17]. MMTV encodes a regulatory protein Rem [13,18] that is functionally equivalent to HIV Rev. Rem interacts with unspliced or singly-spliced RNA through a secondary structure, the Rem response element (RmRE), that spans the 3' end of the *env *gene and part of the 3'long terminal repeat (LTR) [14,19]. This interaction is required for transport of genome-length RNA from the nucleus to the cytoplasm through Crm1 [13,18].

Although initially identified due to their role in transport of unspliced viral RNA from the nucleus to the cytoplasm, regulatory proteins like Rev have been shown to possess other functions. Both HIV-1 Rev and Jaagsiekte sheep retrovirus (JSRV) Rej enhance *gag *translation [15,20]. HIV-1 Rev accomplishes this by increasing the stability of *gag*-containing mRNA in the cytoplasm [21] and by enhancing the association of RRE-containing mRNAs with polysomes [20,22]. The mechanism of translational enhancement by JSRV Rej has not been thoroughly characterized, but is clearly independent of mRNA export [15]. The stability of HIV-1 unspliced mRNAs is regulated by inhibitory or instability sequences (INS) present in introns, including the *gag *ORF. These elements function in the absence of other viral genes or proteins and render the RNA unstable in the cytoplasm such that the presence of INS can result in reduced translation [21]. The RNA instability can be overcome by silent mutagenesis of the INS or by addition of Rev in *trans *[23]. Similarly, Butsch *et al*. have demonstrated that, despite efficient RNA export to the cytoplasm, *gag*-containing constructs fail to translate unless 5'-UTR elements, termed post-transcriptional control elements (PCE) interact with RNA helicase A [24-27]. These data further support the existence of inhibitory sequences within the *gag *ORF.

Relatively little is known about the basic virus-host interactions leading to MMTV expression, assembly and replication in contrast to the current understanding of MLV-, or HIV-1-host interactions. This lack of knowledge fundamentally results from an inability to bacterially propagate MMTV proviral genomes or subviral constructs containing intact *gag *genes derived from exogenous virus but not those from germline-encoded elements. The *gag *genes from endogenous murine viruses propagate in plasmid vectors; therefore, the two available MMTV infectious molecular clones pHyb-Mtv [28] and pGR102 [29] contain the *gag *gene from germline-encoded *Mtv1 *and *Mtv8*, respectively. They differ in the remainder of the viral genome. The sequence that impedes bacterial propagation of MMTV is localized in the pp21 domain of *gag *and has been termed the "bacterial poison sequence" [30]. An exogenous, GR strain *gag *gene, containing a 57-nucleotide deletion in the pp21 domain, efficiently propagated in bacteria, suggesting that the deleted region encompassed the bacterial poison sequence. Zabransky *et al*. silently mutated all the possible nucleotides in the sequence targeted by the deletion creating a Gag-only expression construct SM (silently mutated) that readily propagated as a bacterial plasmid and expressed Gag in eukaryotic cells [31].

However, SM Gag failed to assemble into virus-like particles or into detectable subviral structures *in vitro*, or in cells (unpublished data). Since the *gag *gene in the pHyb-Mtv infectious molecular clone (Mtv-1) makes infectious progeny upon SM Gag expression, it is clear that this *gag *allele is expression and assembly competent. Nevertheless, when Mtv-1 *gag *was introduced into the same expression construct used to successfully express SM Gag, no protein was expressed. Similar results were obtained from a highly homologous, putative human-origin, MMTV *gag*, HBRV (human betaretrovirus) in the same expression system. In contrast, all three *gag *genes expressed equivalently in cell-free, coupled, transcription-translation reactions.

We show that transcript abundance, nuclear-cytoplasmic transport, and transcript stability were equivalent among the inhibited Mtv-1 and HBRV alleles as well as Gag-producing SM allele. Our results suggest that inhibition of *gag *expression is a nuclear event that manifests its phenotype in the cytoplasm through translational inhibition. Interestingly, rescue of the phenotype requires the presence of the RmRE, and for *gag *to be in an intron flanked by splice donor and acceptor sites. Therefore, the history of the mRNA in the nucleus imparts an inhibitory phenotype that can be overcome by Rem and splicing motifs. Our results suggest contributions of splicing motifs and machinery and RNA export signals in the 'licensing' of the MMTV *gag*-containing mRNAs for translation.

## Results

### The silently-mutated *gag *expresses protein from a heterologous promoter, but wild-type Gag expression is undetectable

**Figure 1:**
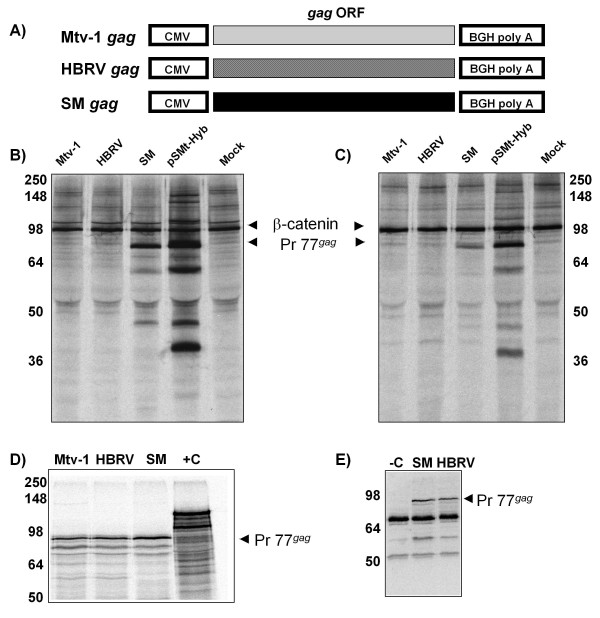
**Gag constructs produce protein *in vitro *but not in mammalian cells**. **A) **Schematic representation of the three *gag *expression constructs: Mtv-1 *gag*, HBRV *gag *and SM *gag*. CMV = human cytomegalovirus immediate early promoter; ORF = open reading frame; BGH polyA = bovine growth hormone polyadenylation site. **B) **HEK 293T cells and **C) **MEF cells were transfected with the indicated *gag *plasmids. Gag protein production was assayed 24 hr post-transfection by metabolic labeling with^35^S methionine (1 hr pulse) followed by immunoprecipitation with rabbit polyclonal anti-MMTV CA sera and an antibody to β-catenin to control for cellular equivalents. Pr77*^gag^*, Gag precursor (77 KDa) and β-catenin (98 KDa) are indicated. **D) **The three *gag *constructs were transcribed and translated *in vitro *in a rabbit reticulocyte lysate. A T7 promoter-driven β-*galactosidase *gene that produces a 110 KDa product was included as a system control (+C). **E) **Western immunoblot of *in vitro *translated Gag with MMTV anti-CA sera. In this case, the β-galactosidase reaction serves as a negative control for the antibody (-C).

Three *gag *alleles were cloned into a cytomegalovirus (CMV) immediate-early promoter vector (pcDNA3.1) that has a bovine growth hormone polyadenylation signal (Figure 1A). The ORFs were introduced to place the *gag *ATG initiation codon into a consensus Kozak sequence, and all constructs were verified by DNA sequencing. The Mtv-1 *gag *was selected because it is plasmid-propagatable and because an infectious molecular clone (pHyb-Mtv) containing this allele exists. A putative human origin MMTV *gag *allele, (HBRV), that is 99.7% identical to Mtv-1 at the nucleic acid and amino acid levels, was also included. The third allele, SM, was derived from the MMTV GR strain, but was previously silently mutated [31] in the *gag *pp21 domain to eliminate the bacterial poison sequence. Despite discrete points of polymorphic sequence, HBRV and Mtv-1 *gag *do not phylogenetically segregate from one another, but both segregate from MMTV GR, the source of the SM *gag *allele. The level of nucleic acid identity between HBRV/Mtv-1 and SM *gag *is 92.7%, whereas the amino acid identity is 95.9%. The highest disparity between HBRV/Mtv-1 and SM lies within the silently mutated *gag *pp21 region. The alterations in SM result in an increase in the overall G/C content in this region from 31.5% to 51.9%, yet none of the three *gag *genes appeared more codon optimized than another as indicated by their codon adaptation indices (Mtv-1 = 0.137, HBRV = 0.138, SM = 0.151, compared to an optima of 0.958) [32].

Gag expression constructs were transfected into HEK 293T cells and subjected to metabolic labelling followed by immunoprecipitation with a rabbit polyclonal anti-MMTV CA antibody. Little or no Mtv-1 Gag or HBRV Gag was detected when expressed from CMV-based *gag*-only constructs; however, SM Gag was readily detected. Moreover, Mtv-1 Gag derived from the pSMt-Hyb provirus was also expressed to detectable levels (Figure 1B). Quantification of the levels of pulse-labeled Gag derived from the CMV vectors revealed an approximately 95-fold inhibition of these two constructs relative to SM Gag (p < 0.05). Expression of another MMTV Gag (Mtv-8) from the same vector was similarly restricted (data not shown). Given that Mtv-1 *gag *and the *gag *from the pSMt-Hyb provirus are identical, these results indicated the presence of a repression acting against Mtv-1 Gag ORF expression as well as a virus-mediated derepression that allows Mtv-1 Gag expression from the provirus.

Since human cells are not predicted to be the natural host for MMTV replication, barriers to MMTV expression could be responsible for the inhibition of Gag expression observed. A similar inhibition has been reported in efforts to express HIV-1 Gag in murine cells [33]. Therefore, we tested whether Gag expression constructs produced protein in C57BL6 murine embryonic fibroblast (MEF) cells. We were surprised to find that both Mtv-1 and HBRV failed to express Gag whereas the pSMt-Hyb infectious molecular clone and the SM Gag variant expressed at high levels (Figure 1C, SM and pSMt-Hyb lanes). MMTV is clearly capable of replication in murine cells, and the virus is readily produced by transfection of infectious molecular clones into human cells [34-36]. Our data suggest a blockade to translation of Gag expressed in the absence of the rest of the genome in murine cells, and these data are supported by earlier reports from Vaidya *et al*., although the cause for this lack of expression was not determined [37].

Gag expression was also tested in two human breast cancer cell lines MCF7 and T47D and in African green monkey kidney (COS-1) cells. Similar to our data for Gag expression in HEK 293T and MEF cells, little or no HBRV or Mtv-1Gag was detected, but SM Gag expression was evident in all cell lines tested (Additional File 1).

### All *gag *alleles express *in vitro*

Given the perplexing lack of protein expression in the CMV-*gag *transfection experiments, we sought to verify construct integrity and the sensitivity and capability of our polyclonal antibody to detect proteins derived from the various *gag *alleles. We took advantage of the fact that pcDNA3.1 vectors contain a phage T7 RNA polymerase promoter that can be used to express RNA *in vitro*. The *in vitro *synthesized RNA was then used to program *in vitro *rabbit reticulocyte lysate translation reactions that included [^35^S]-methionine. The resulting labelled protein was separated by SDS-PAGE and either visualized directly by autoradiography, or subjected to immunoblotting. All Gag constructs expressed similar levels of protein in reticulocyte lysates (Figure 1D), demonstrating that all three mRNAs are competent for programming transcription and translation *in vitro*. We verified these bands were indeed Gag since the product of each construct was equivalently detected by immunoblotting with polyclonal anti-MMTV CA antibody (Figure 1E). These results demonstrate that there is neither differential detection nor differential translation capacity between constructs. Taken together, these results support the concept of specific inhibition of expression of Gag dependent upon the allele present in the construct.

### Expression timing and protein stability do not account for lack of Gag protein

**Figure 2:**
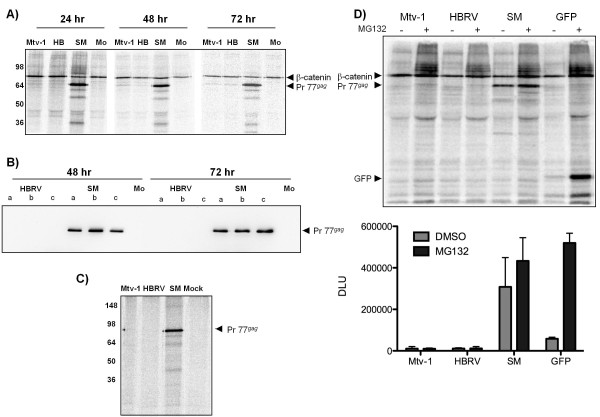
**Lack of Gag expression is not a function of timing or protein stability**. **A) **293T cells were transfected with the indicated *gag *constructs and (24, 48 or 72 hrs post-transfection) were metabolically labelled for 1 hr followed by IP with the MMTV anti-CA antibody and a control antibody to β-catenin. Pr77*^gag^*, Gag precursor. (77 KDa) and β-catenin (98 KDa). **B) **Steady-state levels of Gag in HEK 293T cells at 48 and 72 hrs after transfection were detected by using immunoprecipitation followed by Western blot. Triplicate transfections (a, b, c) are shown, Mo = Mock transfected cells. Pr77*^gag^*, the Gag precursor (77 KDa). **C) **HEK 293T cells were transfected with the indicated plasmids or mock transfected. Twenty-four hours post-transfection, the cells were subjected to a 15-min pulse followed by lysis and immunoprecipitating with the MMTV anti-CA antibody. Pr77*^gag^*, Gag precursor. **D) **HEK 293T cells were transfected with the indicated *gag *constructs or *gfp *and treated 24 hr later with the proteasome inhibitor MG132 for 2 hr. Gag and GFP levels were quantified by radiolabeling and immunoprecipitating with an MMTV anti-CA antibody or with an anti-GFP antibody. Lower panel shows quantification of the Gag and GFP levels in the presence or absence of the proteasome inhibitor. Data are average of three independent experiments ± SD. "+" = MG132-treated cells, "-" = DMSO-treated cells, DLU = digital light units. Pr77*^gag^*, the Gag precursor (77 KDa), β-catenin (98 KDa), and GFP (27 KDa).

Since all three *gag*-only constructs have the same promoter and would be anticipated to express with similar kinetics, we determined the expression of each at 24, 48 and 72 hrs post-transfection by pulse labeling. SM Gag was detectable, but Mtv-1 and HBRV Gag expression was inhibited at all time points (Figure 2A). We also assessed steady-state Gag levels by immunoblotting. Only the SM Gag was detected 48 and 72 hrs post-transfection (Figure 2B). One explanation for the above results might be that Mtv-1 and HBRV Gag are highly labile proteins and therefore do not accumulate. To test this concept, we subjected CMV-*gag *transfected cells to metabolic pulse-labeling for 15 minutes with no chase. This time-frame was anticipated to catch synthesized protein before it could be degraded. Under these conditions, we did not detect either Mtv-1 or HBRV Gag. A robust SM Gag band was evident (Figure 2C). We also used the proteasomal inhibitor MG132 (Figure 2D) or lactacystin (not shown) to block protein degradation and turnover. Proteasomal inhibition was demonstrated by a treatment-induced increase in GFP as well as increased β-catenin signal at 98 KDa. We also noted the presence of higher molecular weight bands in the presence of the proteasomal inhibitor that likely represent accumulation of ubiquitinated and polyubiquitinated β-catenin (Figure 2D). These higher weight forms of β-catenin are also present in the GFP-only lane, and therefore, are not Gag. Despite evidence of proteasome inhibition, no Mtv-1 or HBRV Gag was detected in treated cell lysates. SM levels, however, increased in response to treatment, indicating that a substantial proportion of Gag fails to assemble, and is degraded intracellularly (Figure 2D), as has been demonstrated in other retroviral systems [38]. Thus our results show that Mtv-1 and HBRV *gag *constructs fail to undergo protein synthesis rather than producing a labile gene product, whereas the SM *gag *construct is not subject to a similar constraint.

### Gag transcripts are efficiently synthesized in human cells

**Figure 3:**
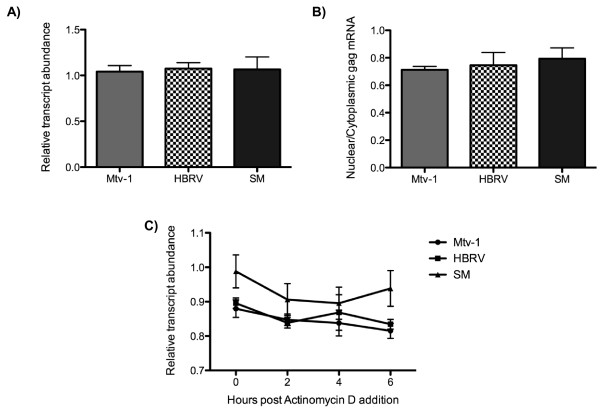
**Inhibition of expression occurs post-transcriptionally and does not affect mRNA transport and stability**. **A) **Total RNA was extracted from HEK 293T cells transfected with Mtv-1, HBRV, and SM *gag *constructs. The *gag *and *gapdh *mRNAs were reverse transcribed into cDNA using an Oligo-dT primer and quantified by Real-Time PCR. The relative abundance of *gag *mRNA to *gapdh *is calculated as the reverse of the ratio of *gag *CT to *gapdh *CT value. **B) **Transfected HEK 293T cells were separated into nuclear and cytoplasmic fractions. The mRNA was extracted from each fraction, and *gag *and *gapdh *mRNAs were quantified by reverse transcription Real-Time PCR. The level of mRNA transport from the nucleus to the cytoplasm was quantified as the ratio of nuclear and cytoplasmic *gag *mRNA relative to *gapdh*. **C) **Cells were treated with actinomycin D, and RNA was extracted from the cells at 0, 2, 4 and 6 hrs post-treatment and quantified by reverse transcription Real-Time PCR. The relative abundance of *gag *mRNA to *gapdh *was calculated at each time point. Data are the average of at least three independent experiments ± SD.

Our data suggest that the lack of detectable Gag results from a lack of Gag protein synthesis and not from protein degradation or instability. Therefore, the block in protein expression could result from three potential defects: the lack of *gag *mRNA, the lack of *gag *mRNA transport to the cytoplasm, or the inability to translate the Gag transcript if synthesized and appropriately transported. To identify the defect, we tested for correlations between *gag *mRNA levels and protein expression between constructs. Real-time PCR (qRT-PCR) was used to quantify transcript abundance, nuclear to cytoplasmic transport, and mRNA stability. We primed cDNA synthesis in transfected cells from an oligo-dT primer and performed Taqman detection using an amplicon over conserved sequences in pp21. The amplification signal (C_t_) for *gag *mRNA from the respective constructs was normalized to that of cellular *gapdh *and the reciprocal ratios were compared. We observed no significant difference in mRNA abundance for the different *gag *alleles (Figure 3A), indicating that all *gag *transcripts were present at the same level and that the lack of Gag expression was not a function of mRNA abundance.

### Nuclear to cytoplasmic transport and mRNA stability are similar for all *gag *alleles

We next asked whether the various *gag *mRNAs can traffic from the nucleus to the cytoplasm. Subcellular fractionation was used to extract mRNA from nuclear and cytoplasmic fractions, and the abundance of the *gag *transcript in each fraction was quantified relative to *gapdh *by using qRT-PCR. To control for proper nuclear and cytoplasmic separation, we assessed the distribution of histone 3 and GAPDH proteins in the different fractions. We found that histone 3 was present only in the nuclear fraction while GAPDH was only in the cytoplasm (data not shown). All *gag *mRNAs were detected in the cytoplasmic fraction, and quantification of the ratio of the nuclear-to-cytoplasmic transcript abundance revealed no difference between *gag *constructs; therefore, the transport of all *gag *mRNAs was equivalent (Figure 3B). This result suggests that Mtv-1 and HBRV Gag expression was not impaired by a defect in mRNA transport.

Inhibited translation of HIV-1 *gag *has been linked to cytoplasmic instability of the RNA imparted by instability (INS) sequences in Gag [21,39]. To determine whether the various *gag *mRNAs displayed differential stability in the cytoplasm, CMV-*gag *transfected cells were treated with the transcription inhibitor actinomycin D. The *gag *mRNA abundance was quantified relative to *gapdh *by using qRT-PCR at 2, 4, and 6 hr after treatment. No differences in mRNA stability were detected among Mtv-1, HBRV and SM over the 6 hr treatment course (Figure 3C).

Since we determined that analyses of RNA synthesis, transport and stability do not affect the differential protein expression phenotypes evinced between SM and the two other MMTV *gag *alleles, we conclude that the block to Gag expression occurred post-transcriptionally such that the mRNA is not translatable although it was transported to the cytoplasm.

### Negative regulation against Mtv-1 and HBRV *gag *acts at the transcript level

**Figure 4:**
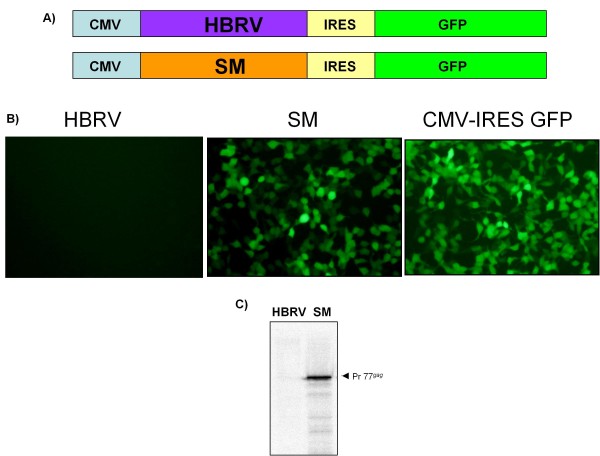
**Negative regulation against Mtv-1 and HBRV *gag *acts at the transcript level**. **A) **The *gag *ORF was cloned in the multiple cloning site of pIRES-eGFP upstream of the IRES and GFP, and was under the control of the CMV promoter. **B) **Expression of GFP was visualized by fluorescence microscopy. The "empty" pIRES-eGFP construct was used as a control for GFP expression. **C) **Gag levels were quantified by pulse labeling and immunoprecipitation with an MMTV anti-CA antibody. Pr77*^gag^*, the Gag precursor (77 KDa).

Post-transcriptional inhibition of Gag expression could function in two ways: (1) the inhibition directly targets the transcript for sequestration or degradation similar to that which occurs in RNAi pathways or (2) Mtv-1 and HBRV *gag *transcripts fail to support translation initiation. In the latter, this would be envisioned as a failure to successfully recruit or engage translation factors essential for cap-dependent translational initiation. To differentiate between these mechanisms, we introduced *gag *alleles into a bicistronic plasmid vector in which the Gag translation is driven by cap-dependent initiation and eGFP is translated from an encephalomyocarditis virus- internal ribosome entry site (EMCV-IRES) (Figure 4A). We reasoned that if inhibition was at the level of *gag *translation initiation, then no Gag would be produced from cap-dependent initiation; but the production of eGFP from the cap-independent IRES would proceed unabated. However, if the inhibition acts to prevent all interactions of the mRNA with translational machinery, then neither cistron would be expressed.

Bicistronic constructs were transfected into HEK293T cells and the transfected cultures were observed by *in situ *fluorescent microscopy for eGFP expression prior to lysis and immunoprecipitation to quantify MMTV Gag expression. In a parallel analysis, we used qRT-PCR to demonstrate that all bicistronic constructs transported mRNA to the cytoplasm (data not shown). We observed no eGFP signal from the bicistronic construct containing HBRV *gag*, and immunoprecipitation revealed no detectable Gag from the first cistron of this construct (Figure 4B and 4C). In contrast, for the SM bicistronic construct both proteins were abundantly produced (Figure 4B and 4C). Since both constructs contained the same Kozak sequence and IRES, the inhibition resulted from recognition of sequences within *gag *and not from a defect in translational initiation. Thus, *gag *sequences appear to either inhibit translational 'licensing' of the mRNA or to completely sequester the mRNA away from translational machinery.

### Inhibition is dependent on the nuclear history of the mRNA

**Figure 5:**
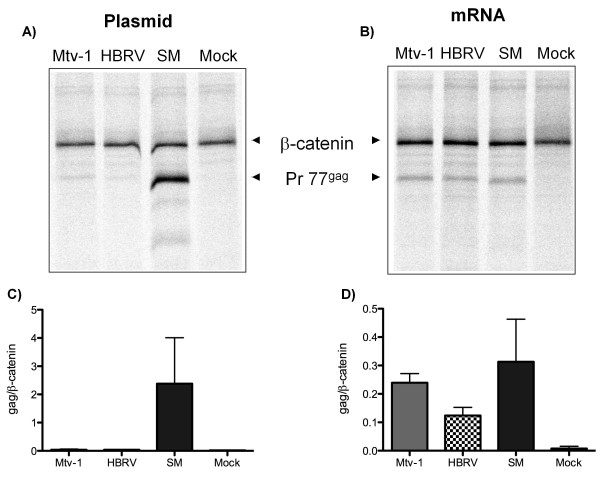
**Inhibition is dependent on the nuclear history of the mRNA**. **A) **Plasmid or **B) ***in vitro *made mRNA for the indicated constructs was transfected into HEK 293T cells. After 24 hr, Gag expression was quantified by pulse labeling for 1 hr and immunoprecipitating with an MMTV anti-CA antibody. β-catenin immunoprecipitation served as a loading control. Gag expression from plasmid **C) **or *in vitro *made mRNA **D) **was quantified relative to β-catenin. Data represent the average of three experiments ± SD. Pr77*^gag^*, the Gag precursor (77 KDa) and β-catenin (98 KDa).

Analysis of bicistronic constructs suggested that the *gag *mRNA was not competent for translation (Figure 4), yet all three *gag *mRNAs can program a rabbit reticulocyte lysate to make protein (Figure 1D). We, therefore, tested whether *in vitro *synthesized *gag *RNAs were competent to program translation if delivered directly to cells by transfection. This approach was also reasoned to provide some capacity to distinguish between cytoplasmic or nuclear mechanisms of translational inhibition. The pcDNA3.1 constructs contain a T7 RNA polymerase promoter that was used to drive RNA synthesis *in vitro*. A portion of each *in vitro *synthesized *gag *RNA was used to program a rabbit reticulocyte lysate to test RNA integrity and the remainder of the same preparation was introduced into HEK293T cells using lipid mediated transfection. In contrast to transfection of plasmid pCDNA3.1 *gag*constructs (Figure 5A and 5C), the *in vitro *synthesized *gag *RNAs (derived from the same construct) were fully competent to program translation in the RNA transfected cells (Figure 5B and 5D). While we do not formally know whether the introduced RNA remained cytoplasmic or trafficked to the nucleus, these results suggest that inhibition of Mtv-1 and HBRV Gag translation was imposed in the nucleus or acts co-transcriptionally.

### Rescue of Gag expression by the *RmRE *and Rem in *trans *is dependent on splice recognition sites

Expression of MMTV *gag *from a proviral construct is readily detected while the same reading frame is inhibited in a *gag*-only construct. This suggests an inhibition targeting *gag *ORF and a viral mechanism to overcome it. We have shown that this inhibition targets the mRNA in the nucleus and prevents its translation in the cytoplasm. From studies using other retroviruses, there are two potential viral mechanisms that rescue Gag expression from inhibition. The first, as exemplified by spleen necrosis virus and Mason-Pfizer monkey virus, involves an interaction between cellular RNA helicase A and the viral 5' UTR that enhanced viral mRNA association with ribosomes [24,25]. The second is post-transcriptional enhancement of HIV-1 translation by the viral Rev protein [40].

#### The MMTV LTR and 5'UTR sequences are not sufficient to rescue Gag expression

The natural promoter for MMTV Gag is in the 5'LTR and requires glucocorticoid stimulation for activity [41]. While expression from the LTR is typically lower than from CMV promoter-driven over-expression systems, testing whether addition of glucocorticoid (dexamethasone) rescued expression was deemed worthwhile. Moreover, given reports that the presence of an authentic 5' UTR can be important for regulating retroviral expression [42], and that the 5'-UTR can enhance translation of the *gag *gene [25], we created an MMTV *gag *construct driven by its own LTR. We deleted *pol *and part of the *env *gene from pHyb-Mtv while maintaining the 3'LTR. The resultant construct, pLTR-*gag *(Figure 7A), contains the 5' and 3' LTRs, Mtv-1 *gag*, *pro*, the *RmRE *and *sag*. Gag expression from this construct was reduced compared with that from the intact provirus (Additional File 2). This result suggests that having all of the known 5' *cis*-sequences, the RmRE and the 3' LTR from the provirus fails to support expression of Gag at levels similar to an intact proviral construct containing the same *gag *ORF. Moreover, addition of dexamethasone had no impact on Gag expression except from the intact provirus. Thus the 5'-UTR appears to be insufficient to rescue Gag expression.

#### Rem and RmRE are insufficient to rescue CMV-driven Gag expression

The *gag *expression constructs presented thus far do not contain known splice sites and thus would be anticipated to be independent of the RNA transport-mediated activities of Rem. Our data quantifying nuclear and cytoplasmic *gag *mRNA levels support this concept. However, several reports have suggested post-transcriptional roles for Rev and its analogs that potentially impact Gag protein expression [15,21]. These data prompted us to test whether the observed restriction against Gag expression results from the lack of Rem or the lack of a Rem/RmRE interaction.

**Figure 6:**
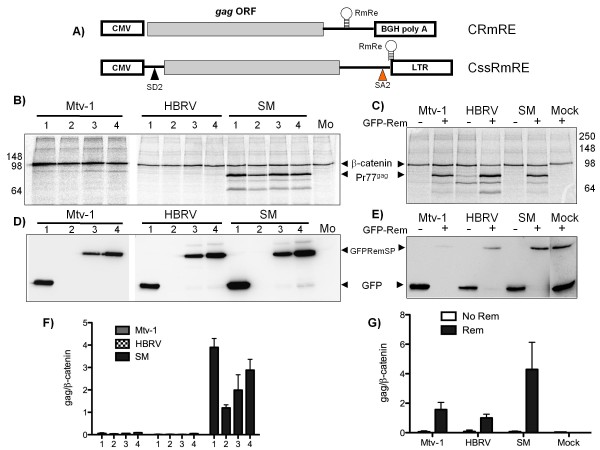
**Rem in *trans *along with RmRE rescues Gag expression from an artificial intron**. **A) **Schematic representation of the *gag *constructs that contain the RmRE. First the RmRE was introduced in the 3'UTR of pcDNA3.1 *gag *construct, downstream of the *gag *stop codon, but upstream of the polyA site (CRmRE). In a second construct *gag *was inserted between the second splice donor and splice acceptor from the MMTV infectious molecular clone, and upstream of the RmRE and the viral 3'LTR (CssRmRE). Mtv-1, HBRV, and SM *gags *from the CRmRE **B) **and CssRmRE **C) **were expressed in HEK 293T cells in the presence or absence of Rem. Lanes indicate 1, GFP; 2, Rem; 3, GFP-Rem; 4, GFP-RemSP; +, GFP-Rem; -, GFP co-transfections. Gag expression was assayed 24 hrs post-transfection by radiolabeling and immunoprecipitating with MMTV anti-CA and anti-β-catenin antibodies. Rem expression in the same samples CRmRE **D) **and CssRmRE **E) **is shown by Western blot with a rabbit polyclonal anti-GFP antibody. Pr77*^gag^*, Gag precursor (77 KDa), β-catenin (98 KDa), GFP-RemSP (40 KDa) and GFP (27 KDa). Gag levels from CRmRE **F) **and CssRmRE **G) **were quantified relative to β-catenin ± SD in the presence and absence of different Rem constructs. Lanes indicate 1, GFP; 2, Rem; 3, GFP-Rem; 4, GFP-RemSP; +, GFP-Rem; -, GFP. Data are average of three independent transfections.

We began by verifying the functionality of three previously published Rem constructs (Rem-only, GFP-Rem, and GFP-RemSP) [13] by testing their capacity to mediate expression of an intronic *Renilla *luciferase reporter from pHMR*luc*, a construct that also contained the RmRE (Additional File 3). We tested whether providing Rem in *trans *altered expression from the CMV-*gag *only constructs lacking a RmRE (not shown) and observed no effect. Thus, the silent alterations in SM *gag *did not introduce a Rem responsive *cis-*element. Next, we cloned the RmRE sequence (nt 7291-7886) into the 3' UTR of the CMV-driven *gag *constructs (Figure 6A). For a precise location of the RmRE sequence compared to the *Mtv-1 *genome and relative to the published RmRE see Additional File 4. After verifying that no extraneous mutations were introduced in *gag*, we demonstrated that the construct expression profiles remained unchanged from those observed in the absence of the RmRE (Figure 6B compare lanes '1' to Figure 1B). However, co-transfection of Rem did not rescue Gag expression despite the presence of the RmRE (Figure 6B and 6F; lanes 2, 3, and 4). In Figure 6D, anti-GFP immunoblotting was utilized to demonstrate expression of the two Rem fusion constructs provided in *trans*. Moreover, no difference in the levels of cytoplasmic mRNA between *gags *expressed in the presence or absence of Rem was detected. This result suggested that providing Rem in *trans *did not alter nuclear-to-cytoplasmic transport of these *gag *mRNAs despite the presence of its cognate RmRE *cis *sequence.

#### Rem in trans along with RmRE rescues Gag expression from an artificial intron

When expressed from the viral LTR, both the genome length *gag*/*gag-pro-pol *mRNA and the subgenomic, singly-spliced *env *mRNA are contained within introns. Yet the cellular default is to completely splice pre-mRNA before transport to the cytoplasm. Like other complex retroviruses, MMTV encodes a *trans*-acting, Rev-like regulatory protein, Rem, which in combination with the RmRE, is necessary for expression of intronic viral gene products [13,18]. We replaced *Renilla luciferase *in pHMR*luc *gene with the three *gag *genes. In this context, *gag *is in the intron and should not be expressed unless splicing is overcome by a transport regulator. As anticipated, no Gag expression was detected from this construct in the absence of Rem (Figure 6C and 6G '-' lanes). However, with Rem in *trans*, equivalent Gag expression was evident for all three constructs (Figure 6C and 6G**'+' **lanes). Figure 6E shows immunoblots of the GFP-Rem fusion proteins and controls. Results from these investigations demonstrate that Rem can rescue Gag expression but only when *gag *is flanked by a splice donor and acceptor, and the mRNA contains the RemRE.

#### Rem along with the RmRE and natural splice donor and acceptor sequences rescues Gag expression

**Figure 7:**
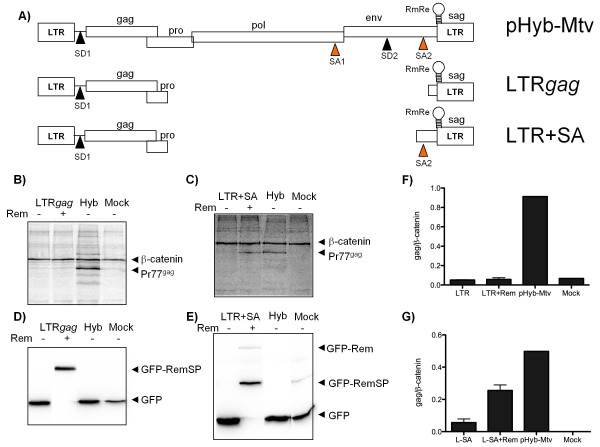
**Rem, the RmRE and natural splice donor and acceptor sequences rescue Gag expression**. **A) **Schematic representation of the MMTV infectious molecular clone (pHyb-Mtv) and the two subviral clones created (LTR*gag *and LTR+SA). LTR, long terminal repeat; SD, splice donor; SA, splice acceptor, RmRE, Rem response element. *gag*, *pro*, *pol*, *env*, *sag *are the viral genes. Expression of Gag from pLTR*gag ***B) **or pLTR*+*SA **C) **was assayed in 293T cells by pulse labeling and immunoprecipitating with an MMTV anti-CA antibody in the presence or absence of GFP-Rem. Transfection of pHyb-Mtv served as a positive control. '-' lanes were transfected with GFP to equilibrate the plasmid load. β-catenin was used as a cellular control. Expression of GFP and GFP-Rem was visualized by immunoblotting with an anti-GFP antibody in pLTR*gag ***D) **or pLTR*+*SA **E) **transfected cells. Pr77*^gag^*, the Gag precursor (77 KDa), β-catenin (98 KDa), GFP-Rem (66 KDa), GFP-RemSP (40 KDa) and GFP (27 KDa). Gag levels from the LTR*gag ***F) **and LTR*+*SA **G) **were quantified relative to β-catenin. Data are average of three independent transfections ± SD.

Based on the results demonstrating Gag expression from an artificial intron RmRE construct, we revisited the MMTV pLTR-*gag *construct where Rem failed to rescue Gag expression despite the fact that pLTR-*gag *contained an intact RmRE (Figure 7B and 7F and Additional File 4). As in Figure 6, immunoblotting for GFP was utilized to demonstrate expression of the GFP-Rem fusion protein (Figure 7D and 7E). Although the major splice donor was present in pLTR-*gag*, both viral splice acceptors had been deleted (Figure 7A). Upon reconstruction of the splice acceptor into the pLTR-*gag *construct (pLTR-*gag*SA), Rem was able to rescue Gag expression (Figure 7C and 7G and Additional File 4). This result further supports the concept that Rem requires, in addition to the RmRE, the recruitment of splicing machinery to *gag *intron splice donor and acceptor sites, yet such recruitment does not result in cleavage of the intron. This complex of *cis *and *trans *factors is necessary to support MMTV Gag translation.

## Discussion

Nuclear protein-mRNA interactions impact viral RNA transport. The transport mechanisms (Crm1 or Tap/NXT), in turn, influence the localization and fate of the viral mRNA or gRNA in the cytoplasm. These relationships are evident from studies of RSV in which deletion of a direct repeat upstream of *src *that functions as a CTE leads to efficient Gag production but lack of particle assembly [43,44]. Similar defects are observed in attempts to express ALV in mammalian cells [45-47], but the defect can be overcome by driving ALV through the Crm1 RNA export pathway by providing an HIV-1 RRE and Rev *in trans *[48]. HIV-1 Gag expression can be achieved in murine cells, but processing, viral RNA encapsidation, and particle formation are not supported unless the HIV-1 RNA is exported via a CTE export pathway [33]. Murine expression of HIV-1 Gag can be achieved in a Rev/RRE/Crm1-dependent pathway but only if human SRp40 and SRp55 are provided ectopically [49]. In addition, post-transcriptional control elements (PCE) in the 5' UTR of a number of retroviruses have been shown to interact with nuclear or cytoplasmic RNA helicase A to promote mRNP remodeling that facilitates polyribosome association resulting in efficient translation [26,42]. Thus protein marks applied to the pre-mRNA in the nucleus, in addition to defining the RNA export pathway, contribute to localization and fate of the RNA in the cytoplasm.

From the studies mentioned above, as well as from our CMV-*gag *expression data (Figure 1), it is clear that mRNA export alone is insufficient to stimulate translation of retroviral Gag. All alleles of MMTV *gag *that were tested with the CMV promoter were efficiently exported, presumably by a Tap/NXT pathway, yet differential Gag translation occurred among the alleles. We propose that RNA 'fate' determinants are being applied to MMTV *gag *mRNAs that are dependent upon sequences within the ORF. These RNA fate decisions are potent enough to prevent cap-independent translation of reporters in the second position of bicistronic constructs, suggesting that nuclear 'marks' applied to the mRNA and direct its cytoplasmic localization such that it is sequestered from all translation machinery. Yet, the RNA is not degraded.

Rev controls the transition between early and late phases of HIV-1 replication by regulating the export of incompletely spliced viral mRNA to the cytoplasm. While promoting unspliced mRNA export is regarded as its primary function, Rev has also been shown to affect the stability, translation and encapsidation of RRE-containing RNAs [40,50]. Rev has been shown to overcome inhibitory or instability (INS) sequences present in the *gag *ORF [21] by preventing cytoplasmic RNA degradation. The *gag *RNA can also be protected from degradation and efficiently expressed if it is codon optimized or if the INS sequences are mutated [39]. The fact that silent mutations in MMTV SM *gag *overcome inhibition of expression acting against Mtv-1 suggests that wild-type MMTV *gag *genes may contain an INS. However, in contrast to the HIV-1 inhibitory sequences, which trigger the viral RNA for degradation, all MMTV *gag *mRNAs were stable in the cytoplasm whether translated or not. Moreover, our analyses of the three MMTV *gag *alleles did not reveal evidence of codon optimization, or lack thereof, by any particular *gag*. Thus, we concluded that MMTV *gag *sequences were subject to post-transcriptional control mechanisms, but they function differently from the mRNA degradation pathways that act against HIV-1 *gag*. HIV-1 INS sequences were bound in the nucleus by PSF, a transcription/splicing factor, which in turn affected the fate of the RNA in the cytoplasm [51]. It remains to be tested whether a similar interaction dictates the fate of the MMTV *gag *alleles.

**Figure 8:**
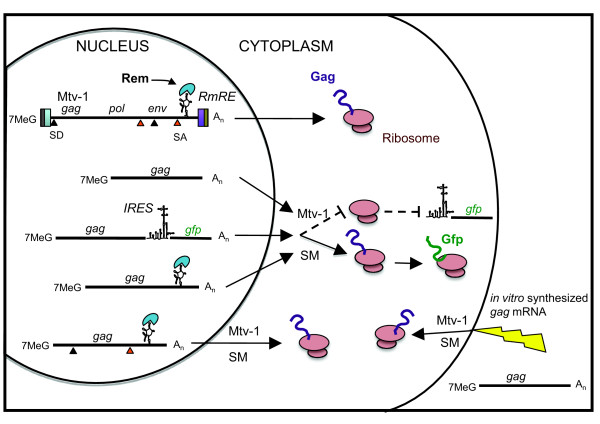
**Schematic representation of *gag *mRNAs and their translation phenotypes**. Full length viral mRNA is capped (7MeG) and polyadenylated (A_n_), as are all other constructs. At the 5' and 3' ends of the viral genome length RNA, the R and U5 or U3 elements are indicated as grey (R) blue (U5) and purple boxes (U3). The viral open reading frames (ORF) *gag*, *pol*, and *env *are indicated while other genes are not presented to maintain clarity. The splice donors (SD) and splice acceptors (SA) are represented as green or orange triangles, respectively. The viral protein Rem (blue half moon) is shown bound to its *cis *RNA structural element, the RmRE. Three subviral mRNAs, derived from various CMV promoter constructs, are also depicted. The minimal expression construct contains only the cap, *gag *ORF, and poly-A tail. Also depicted is the mRNA from a *gag *bicistronic construct that contains an IRES element supporting cap-independent translation of eGFP. The second subviral construct includes the RmRE, to which Rem (provided in *trans*) is shown to bind. The third subviral *gag *expression construct contains both a SD and SA positioning the *gag *ORF in an intron as well as the RmRE and Rem in *trans*. Expression of Gag protein from the different mRNAs is indicated as a purple polypeptide exiting the ribosome (red). For subviral constructs, loss of protein expression as a result of the included *gag *allele is indicated by dotted lines and by the absence of Gag polypeptide exiting the ribosome. Similarly, when the bicistronic constructs fail to support Gag expression, the lack of GFP production from the IRES is indicated by the dotted line and lack of the green polypeptide exiting the ribosome. The lightning bolt represents direct transfection of *gag *mRNAs where allele-independent Gag translation was detected.

Like HIV-1, MMTV has been characterized as a complex retrovirus due to the presence of the Rev analog, Rem, that temporally controls MMTV gene expression [13]. Rem has been shown to interact with a structured element in unspliced and singly-spliced mRNAs [14] and to promote transport of these RNAs to the cytoplasm [13,18]. Mertz *et al*. demonstrate that Rem enhances translation of an intronic reporter without affecting RNA transport [52]. Moreover, Hofacre *et al*. have shown that the function of JSRV Rej appears to be involved in cytoplasmic enhancement of translation not in nuclear-to-cytoplasmic RNA transport [15]. In our hands, *gag *bicistronic constructs were efficiently transported to the cytoplasm. If cap-dependent translation was inhibited by the *gag *allele in the first cistron, no reporter was expressed by cap-independent translation from the second cistron. Thus, the RNA has been targeted by an inhibition that manifests as a total and irreversible block to translation. This inhibition is likely a function of RNA localization as opposed to failure to initiate translation since IRES-mediated translation is also inhibited. These results reinforce the concept that it is the *gag *sequence itself that is the target of inhibition since the eGFP reporter is efficiently co-expressed when SM Gag is expressed. Moreover, direct RNA transfections reveal that the inhibition is nuclear in origin since all *gag *alleles were equally competent to program translation. These allele-specific effects on Gag translation are summarized in Figure 8. We reasoned that the directly transfected RNA either does not have access to the nucleus or it escapes a binding partner that is co-transcriptionally acquired. While our data are consistent with that of others demonstrating a phenotypic translational block, we show that this blockade is actually imposed as a nuclear mark on the RNA.

The nucleotide sequences among complex retroviruses are not highly conserved. It is therefore not surprising that there is little consensus in RNA regulatory mechanisms other than a requirement for a *cis *regulatory element (like the RmRE) and a cognate *trans*-RNA-binding factor (like Rem). Other sequences in the RNA may be essential because of their capacity to specifically coordinate nuclear proteins. In the elucidation of Rem and Rej function, reporter constructs that contain splice sites have been used to assay effects on RNA transport [13,15]. However, in the absence of Rem and Rej the intron-containing constructs were exported, but not expressed. The interpretation was that the function of Rem and Rej was to enable translation rather than RNA transport. The potential role of the splice sites and their capacity to coordinate nuclear proteins that impact mRNA fate was not directly addressed. We have shown that the presence of splice sites, but not their utilization, is required in addition to the RmRE and Rem for Gag expression. This is potentially due to interaction of these sequences with protein partners as has been shown in HIV-1 where U1 snRNP interaction with the 5' splice site is required for Rev function [53]. However, we show that the 5' MMTV splice site alone is insufficient to support efficient export and translation. Similarly, any combination other than a functional splice donor and acceptor with a functional Rem and RmRE interaction is insufficient. Figure 8 shows our conceptual model of Gag translation competence. Retroviral RNAs have been demonstrated to interact with a variety of proteins that are constituents of RNA splicing transport and localization complexes, including hnRNPA1, hnRNP A2, I/PTB, DDX1, DDX3, RHA, nucleolin, Sam68, ASF/SF2, SRp40/p55, EF1α, Pur1α, and Staufen [54-56]. We conclude that MMTV Gag expression requires splicing sites on the mRNA in addition to viral RNA regulatory components. Yet while these interactions are essential to facilitate the cytoplasmic expression of Gag, they do not function normally since the intron is not removed. It is possible that Rem is participating in direct and perhaps inhibitory interactions with splicing components that are important for transport or RNA localization and utilization or perhaps the virus has adapted to subvert nuclear RNA-binding protein function in order to achieve its expression program. Thus, it is clear that in the presence of a complete set of viral *cis *signals as well as the viral Rem protein, MMTV *gag *RNA is exported through Crm1 [13], and through these signals, allele-specific inhibitory mechanisms are countered making *gag *competent for translation. Our findings suggest that continued investigation of the proteins that differentially interact with Mtv-1 and SM *gag *alleles may enlighten our understanding of how nuclear mRNP interactions participate in regulating RNA localization and fate.

## Conclusions

Nuclear interactions inhibit MMTV *gag *gene expression independent of RNA transport, stability, and translation. To overcome the inhibition and achieve translation of MMTV *gag *mRNA in the cytoplasm, both the Rem protein and its cognate binding target the RmRE, and surprisingly, a splice donor and acceptor sequence are required.

## Methods

### Cell culture and transfections

Human embryonic kidney (HEK 293T) cells, and murine embryonic fibroblasts (MEF) were maintained in Dulbecco's Modified Eagle Medium supplemented with 10% fetal bovine serum, L-glutamine (20 mM), penicillin (100 U/mL) and streptomycin (100 μg/mL). Cells were grown to 60-80% confluency in 60 mm plates and were transfected with 5 μg of the indicated plasmid DNA at a 1:3 ratio of DNA to FuGene 6 (Roche), following the manufacturer's instructions and were assayed 24 hr post-transfection, or as indicated.

### Plasmid constructs

All restriction enzymes were purchased from New England Biolabs, and *Pfu *polymerase was acquired from Stratagene. The hybrid MMTV provirus (pHyb-Mtv) (graciously provided by Jackie Dudley Ph.D.) [28] and pSMt-HYB [57], which is an MMTV infectious molecular clone driven by the Mason-Pfizer monkey virus LTR were used as positive controls. The *gag *sequences utilized as templates for amplification, mutagenesis and expression were *Mtv*-1 (accession # AF228550), MMTV GR [58-60] and HBRV [61]. Sequences encoding the *Gag *ORF were PCR amplified using primers: Forward: 5'-ACCATGGGGGTCTCGG-3' and Reverse: 5'- TTACAAGTTTTTTGAATTTTCGG-3'. For HBRV *gag*, the template DNA was amplified by PCR 'stitching' of three patient-derived template fragments,: 174-1 (accession # AF513918), 175-3 (accession # AF513919) and 168-2 (accession # AF513914) [61]. The pHyb-Mtv plasmid was used as a source for Mtv-1 *gag *and the silently-mutated (SM) MMTV GR *gag *from the pDAB-MMTV plasmid, which allows plasmid propagation [31], was the source for SM *gag*. Amplified products were TA-cloned into pCR2.1 TOPO (Invitrogen) and then transferred into the eukaryotic expression vector pcDNA3.1 (Invitrogen) by *Eco*RI restriction digestion and ligation. The orientation of the gene in pcDNA3.1 was determined by *Stu*I restriction digestion, and correctly oriented clones were sequenced across the reading frame to assure no unforeseen mutations occurred during PCR cloning.

The RmRE sequence [14,19] was PCR amplified with the primers: Forward: 5'-TTTGATATCCATTGTTTTCCAGTGCCTTGC-3' and Reverse: 5'-TTTCTCGAGCT CTTTCTATTTTCTATTCCCATTTC-3' and introduced into the *EcoR*V and *Xho*I sites of the pcDNA *gag *constructs. The plasmids pHMRluc, Rem, RemGFP and RemSPGFP were a gift from Dr Jackie Dudley [13]. To introduce the *gag *ORFs in the pHMRluc construct, *Renilla luciferase *was excised by PCR, and the *Xmn*I site reconstituted. The three *gag *ORFs were introduced by digestion and blunt ligation from pcDNA3.1 constructs.

pLTR-*gag *was derived from pHyb-Mtv by excision of the sequence present between the two *Bgl*II sites. To introduce the splice acceptor (SA) site back into the pLTR-*gag*, the sequence spanning the SA (nt 6993-7384) was PCR amplified from pHyb-Mtv using primers that contain the *Bgl*II site (Forward: 5'- AGGAGATCTGCAAATTATGATTTTATCTGCG- 3' Reverse: 5'- CGGCATTTCCCCCTTTTTTC-3'). After PCR, the sequence was introduced into pLTR-*gag *by restriction digestion and ligation. To create bicistronic constructs the three *gags *were cloned in the multiple cloning site (*EcoR*I) of pIRES2-EGFP (Clonetech).

### Immunoprecipitation and Western blot

Cells were washed 3 times with PBS and labeled for 1 hr with^35^S Cys/Met *trans*-label (MP biomedicals) at 100 μCi/mL in Cys/Met-free medium. Immunoprecipitations were performed as described [62]. Briefly, cells were lysed in Lysis Buffer A (0.15 M NaCl, 50 mM Tris-HCl (pH 7.5), 1% Triton X-100 and 1% deoxycholate), the nuclei were removed by centrifugation at 14,000 × g for 1 min, after which SDS was added to 0.1% final concentration. Gag proteins were precipitated with 3 μL rabbit polyclonal anti-MMTV CA antibody and 25 μL formalin-fixed *Staphylococcus aureus*. Immunoprecipitated proteins were separated on 10% SDS-PAGE followed by quantitative phosphorimager analysis. To normalize for quantification, we immunoprecipitated in parallel with an anti-β-catenin antibody (AC-15) (Sigma). The same anti-MMTV CA antibody was used for Western blots. To detect Rem-GFP fusions and GFP we used a rabbit polyclonal anti-GFP antibody (Sigma).

### *In vitro *transcription and translation

To synthesize proteins *in vitro *we used the STP3 kit (Novagen) following the manufacturer's instructions. To program mRNA synthesis, 500 μg of plasmid containing the T7 promoter (pcDNA3.1 Gag or controls) was introduced into a T7 polymerase transcription mixture. The reticulocyte lysate was added to the transcription reaction to translate protein in the presence of^35^S-Methionine. A β-galactosidase plasmid provided with the kit was used as a positive control.

### Protein stability

HEK 293T cells were transfected as described. After 24 hours the cells were pulsed for 15 minutes with 100 μCi^35^S trans label, and the proteins were immunoprecipitated and analyzed by Western blotting with the anti-MMTV CA antibody.

Twenty-four hours after transfection, parallel cultures of HEK293T cells were treated with 20 μM MG132 (Sigma) or an equivalent volume of DMSO for 2 hours to block proteasomal degradation. Concomitantly, cells were metabolically labelled with 100 μCi/mL^35^S Cys/Met *trans*-label (MP biomedicals) in Cys/Met-free medium. After two hours of treatment and labelling, protein accumulation was quantified as described previously.

### RNA quantification

RNA was extracted using Trizol reagent (Invitrogen) following the manufacturer's instructions. Carry-over DNA was digested with TurboDNase (Ambion) 1 hr at 37°C. For cDNA production, 5 μg of RNA were used to template SuperScript III reverse transcription (Invitrogen). RNA abundance was quantified by real-time PCR using Taqman technology on an ABI 7500 Fast Real-time PCR System. Briefly, 20 μL reaction volumes contained 10 μL of 2× Reaction Mixture (Ambion), 1 μL primer and probe mix, 2 μL of the template cDNA and 7 μL water. Standard thermal cycling and data collection parameters were utilized. Amplification of *gag *cDNA was normalized to *gapdh*. Gag primers were forward: 5'-TGAAGAAAAGGAGAAGGCA GA-3', and reverse: 5'-CTCAGGGGACAGGTCATCAT-3' and the probe was 5'-Fam-AAGGCCTTTTTA GCCACAGATTGG-Tamra-3' and GAPDH primers were primer forward: 5'-GAAGGTGAAGGTCGGAGTC-3', and reverse: 5'-GAAGATGGTGATGGGATTTC-3' and probe was 5'-JOE-CAAGCTTCCCGTTCTCAGCC-Tamra -3'.

### Nuclear-cytoplasmic extracts

HEK 293T cells were partitioned into nuclear and cytoplasmic fractions using a Cell Fractionation Buffer (PARIS kit, Ambion). RNA was extracted and quantified as described above. mRNA transport was measured as the nuclear to cytoplasmic *gag *mRNA ratio relative to *gapdh *as measured by using qRT-PCR of cDNA.

### RNA stability

Transcription was blocked by addition of 5 μg/mL actinomycin D. RNA was extracted at 0, 2, 4 and 6 hr after treatment and quantified by using qRT-PCR as described above. The relative transcript abundance at each time point was calculated as the ratio of *gag *CT to *gapdh *CT.

### Fluorescence microscopy

To visualize eGFP expressed from the bicistronic constructs, in transfected HEK 293T cells we used an Olympus IX70 microscope with a DP70 camera at a magnification of 20×.

### *In vitro *synthesized mRNA

To make capped mRNA *in vitro *we used the mMessage mMachine system (Ambion) following manufacturer's instructions. The mRNA was first verified *in vitro *to support translation. Equivalent amounts of mRNA were then transfected into 293T cells using Lipofectamine 2000 (Invitrogen). Gag expression was assayed as described above by pulse labeling and immunoprecipitation.

## List of abbreviations

MMTV: mouse mammary tumor virus; ORF: open reading frame; RmRE: Rem response element; mRNP: messenger ribonucleoprotein; MLV: murine leukaemia virus; CTE: constitutive transport element; HIV-1: human immunodeficiency virus; LTR: long terminal repeat; JSRV: Jaagsiekte sheep retrovirus; INS: instability or inhibitory sequence; PCE: post-transcriptional control element; SM: silently mutated; HBRV: human betaretrovirus; CMV- cytomegalovirus; MEF: murine embryonic fibroblasts; COS-1: African green monkey kidney cells; HEK 293T: human embryonic kidney cells; qRT-PCR: quantitative reverse-transcription polymerase chain reaction; ECMV-IRES: encephalomyocarditis virus internal ribosome entry site; SD: splice donor; SA: splice acceptor.

## Competing interests

The authors declare that they have no competing interests.

## Authors' contributions

IB conducted all experiments, assisted in data interpretation, and prepared the draft version of the manuscript. MS participated in early phases of experimental conceptualization and assisted in data interpretation. JW developed the experimental plan and data interpretations as well as assisting in manuscript preparation and editing. All authors read and approved the final manuscript.

## Additional methods

The human breast cancer cell lines T47D and MCF7 were grown in RPMI-1640 Medium and Eagle's Minimum Essential Medium, respectively, and supplemented with 10% fetal bovine serum, L glutamine (20 mM), penicillin (100 U/mL), streptomycin (100 μg/mL) and 0.01 mg/mL bovine insulin. COS-1 cells were grown in DMEM.

### Luciferase assay

Cells were seeded at 5 × 10^4 ^per well in 24-well plates, then transfected with 2 μg of the appropriate plasmid DNA using Fugene 6 (Roche) at 1:3 DNA:Fugene ratio. Twenty-four hrs after transfection, the cell lysates were assayed for luciferase activity using the *Renilla *Luciferase Assay System (Promega) following manufacturers instructions. Luminescence was integrated over 1 second with a 2-second delay on a PerkinElmer 1420 Multilabel Counter Victor3V. Each transfection was done in triplicate and each lysate was assayed in triplicate.

## Supplementary Material

Additional file 1 **(PPT) Similar Gag expression profiles are seen in several cell types**. The indicated *gag *constructs were transfected into COS-1 cells, and the human breast carcinoma cell lines, T47D and MCF7. After 24 hr Gag expression was assayed by metabolic labeling followed by immunoprecipitating with anti-MMTV CA antibody. +C, pSMt-HYB. Pr77*^gag^*, the Gag precursor (77 KDa) is indicated by the arrow. Click here for file

Additional file 2 **(PPT) The cognate 5' UTR does not rescue Gag expression**. MMTV Gag was expressed in HEK 293T cells either from an intact provirus (pHyb-Mtv) or from the MMTV pLTR-*gag *construct in the presence or absence of dexamethasone **(**Dex). After 24 hrs, dexamethasone was added to the cells and 48 hrs post transfection, cells were radiolabeled and Gag expression was assayed by immunoprecipitating with anti-MMTV CA antibody. Pr77*^gag^*, the Gag precursor (77 KDa). Click here for file

Additional file 3 **(PPT) Rem enhances expression of a reporter gene**. To verify Rem function, we used the reporter plasmid pHMR*luc *in which *Renilla luciferase *is in an intron, upstream of the Rem response element. pHMR*luc *was cotransfected with the indicated plasmids and luciferase activity was integrated over one second. The average of triplicate luciferase readings of three independent transfections ± SD is shown. A plasmid expressing Renilla luciferase from a CMV promoter was used as a positive control (Renilla) and mock-transfected cells were used as a negative control. The GFP lane shows the basal luciferase expression from the pHMR*luc *plasmid. Click here for file

Additional file 4 **(PPT) Location of RmRE elements in constructs**. The viral genomic RNA from nt 6709-8564 in Mtv-1 (see Methods for accession #) is shown on the top line with the splice acceptor 2 (SA2) indicated by the orange triangle and the initiation and termination codons of Sag and Env. The two published RmRE sequences are depicted relative to their position versus the reference genome. Following the published RmRE sequences are those utilize in the current manuscript beginning with the sequence that was introduced into the CMV promoter Gag-only constructs (CRmRE) and the same construct containing splice donor and acceptor sequences (CssRmRE). The final two sequences are those that were introduced into proviral derived Gag expression constructs that lacked the splice acceptor (LTRgag) or contained a functional splice acceptor (LTR+SA). For clarity, promoter and *gag *sequences 5' to the area introduced to contain the RmRE and splice sites are not shown. Click here for file

## References

[B1] Kohler A Hurt E Exporting RNA from the nucleus to the cytoplasm Nat Rev Mol Cell Biol 2007 8 761 773 10.1038/nrm2255 17786152

[B2] Moore MJ Proudfoot NJ Pre-mRNA processing reaches back to transcription and ahead to translation Cell 2009 136 688 700 10.1016/j.cell.2009.02.001 19239889

[B3] Zhong XY Wang P Han J Rosenfeld MG Fu XD SR proteins in vertical integration of gene expression from transcription to RNA processing to translation Mol Cell 2009 35 1 10 10.1016/j.molcel.2009.06.016 19595711 PMC2744344

[B4] Kress TL Yoon YJ Mowry KL Nuclear RNP complex assembly initiates cytoplasmic RNA localization J Cell Biol 2004 165 203 211 10.1083/jcb.200309145 15096527 PMC2172032

[B5] Stoltzfus CM Synthesis and processing of avian sarcoma retrovirus RNA Adv Virus Res 1988 35 1 38 2852891 10.1016/s0065-3527(08)60707-1

[B6] Arrigo S Beemon K Regulation of Rous sarcoma virus RNA splicing and stability Mol Cell Biol 1988 8 4858 4867 2850470 10.1128/mcb.8.11.4858-4867.1988 PMC365579

[B7] Katz RA Kotler M Skalka AM cis-acting intron mutations that affect the efficiency of avian retroviral RNA splicing: implication for mechanisms of control J Virol 1988 62 2686 2695 2839694 10.1128/jvi.62.8.2686-2695.1988 PMC253701

[B8] Katz RA Skalka AM Control of retroviral RNA splicing through maintenance of suboptimal processing signals Mol Cell Biol 1990 10 696 704 2153921 10.1128/mcb.10.2.696-704.1990 PMC360868

[B9] Gruter P Tabernero C von Kobbe C Schmitt C Saavedra C Bachi A Wilm M Felber BK Izaurralde E TAP, the human homolog of Mex67p, mediates CTE-dependent RNA export from the nucleus Mol Cell 1998 1 649 659 10.1016/S1097-2765(00)80065-9 9660949

[B10] Hadzopoulou-Cladaras M Felber BK Cladaras C Athanassopoulos A Tse A Pavlakis GN The rev (trs/art) protein of human immunodeficiency virus type 1 affects viral mRNA and protein expression via a cis-acting sequence in the env region J Virol 1989 63 1265 1274 2783738 10.1128/jvi.63.3.1265-1274.1989 PMC247823

[B11] Malim MH Hauber J Le SY Maizel JV Cullen BR The HIV-1 rev trans-activator acts through a structured target sequence to activate nuclear export of unspliced viral mRNA Nature 1989 338 254 257 10.1038/338254a0 2784194

[B12] Neville M Stutz F Lee L Davis LI Rosbash M The importin-beta family member Crm1p bridges the interaction between Rev and the nuclear pore complex during nuclear export Curr Biol 1997 7 767 775 10.1016/S0960-9822(06)00335-6 9368759

[B13] Mertz JA Simper MS Lozano MM Payne SM Dudley JP Mouse mammary tumor virus encodes a self-regulatory RNA export protein and is a complex retrovirus J Virol 2005 79 14737 14747 10.1128/JVI.79.23.14737-14747.2005 16282474 PMC1287593

[B14] Mertz JA Chadee AB Byun H Russell R Dudley JP Mapping of the functional boundaries and secondary structure of the mouse mammary tumor virus Rem-responsive element J Biol Chem 2009 284 25642 25652 10.1074/jbc.M109.012476 19632991 PMC2757966

[B15] Hofacre A Nitta T Fan H Jaagsiekte sheep retrovirus encodes a regulatory factor, Rej, required for synthesis of Gag protein J Virol 2009 83 12483 12498 10.1128/JVI.01747-08 19776124 PMC2786725

[B16] Cardiff RD Kenney N Mouse mammary tumor biology: a short history Adv Cancer Res 2007 98 53 116 17433908 10.1016/S0065-230X(06)98003-8

[B17] Kinyamu HK Archer TK Modifying chromatin to permit steroid hormone receptor-dependent transcription Biochim Biophys Acta 2004 1677 30 45 15020043 10.1016/j.bbaexp.2003.09.015

[B18] Indik S Gunzburg WH Salmons B Rouault F A novel, mouse mammary tumor virus encoded protein with Rev-like properties Virology 2005 337 1 6 10.1016/j.virol.2005.03.040 15914215

[B19] Mullner M Salmons B Gunzburg WH Indik S Identification of the Rem-responsive element of mouse mammary tumor virus Nucleic Acids Res 2008 36 6284 6294 10.1093/nar/gkn608 18835854 PMC2577329

[B20] D'Agostino DM Felber BK Harrison JE Pavlakis GN The Rev protein of human immunodeficiency virus type 1 promotes polysomal association and translation of gag/pol and vpu/env mRNAs Mol Cell Biol 1992 12 1375 1386 1545819 10.1128/mcb.12.3.1375-1386.1992 PMC369571

[B21] Schwartz S Felber BK Pavlakis GN Distinct RNA sequences in the gag region of human immunodeficiency virus type 1 decrease RNA stability and inhibit expression in the absence of Rev protein J Virol 1992 66 150 159 1727477 10.1128/jvi.66.1.150-159.1992 PMC238270

[B22] Perales C Carrasco L Gonzalez ME Regulation of HIV-1 env mRNA translation by Rev protein Biochim Biophys Acta 2005 1743 169 175 10.1016/j.bbamcr.2004.09.030 15777852

[B23] Schneider R Campbell M Nasioulas G Felber BK Pavlakis GN Inactivation of the human immunodeficiency virus type 1 inhibitory elements allows Rev-independent expression of Gag and Gag/protease and particle formation J Virol 1997 71 4892 4903 9188551 10.1128/jvi.71.7.4892-4903.1997 PMC191719

[B24] Butsch M Hull S Wang Y Roberts TM Boris-Lawrie K The 5' RNA terminus of spleen necrosis virus contains a novel posttranscriptional control element that facilitates human immunodeficiency virus Rev/RRE-independent Gag production J Virol 1999 73 4847 4855 10233946 10.1128/jvi.73.6.4847-4855.1999 PMC112528

[B25] Hull S Boris-Lawrie K RU5 of Mason-Pfizer monkey virus 5' long terminal repeat enhances cytoplasmic expression of human immunodeficiency virus type 1 gag-pol and nonviral reporter RNA J Virol 2002 76 10211 10218 10.1128/JVI.76.20.10211-10218.2002 12239296 PMC136562

[B26] Hartman TR Qian S Bolinger C Fernandez S Schoenberg DR Boris-Lawrie K RNA helicase A is necessary for translation of selected messenger RNAs Nat Struct Mol Biol 2006 13 509 516 10.1038/nsmb1092 16680162

[B27] Bolinger C Sharma A Singh D Yu L Boris-Lawrie K RNA helicase A modulates translation of HIV-1 and infectivity of progeny virions Nucleic Acids Res 38 1686 1696 10.1093/nar/gkp1075 PMC2836548 20007598

[B28] Shackleford GM Varmus HE Construction of a clonable, infectious, and tumorigenic mouse mammary tumor virus provirus and a derivative genetic vector Proc Natl Acad Sci USA 1988 85 9655 9659 10.1073/pnas.85.24.9655 2849114 PMC282828

[B29] Salmons B Groner B Calberg-Bacq CM Ponta H Production of mouse mammary tumor virus upon transfection of a recombinant proviral DNA into cultured cells Virology 1985 144 101 114 10.1016/0042-6822(85)90309-5 2998037

[B30] Brookes S Placzek M Moore R Dixon M Dickson C Peters G Insertion elements and transitions in cloned mouse mammary tumour virus DNA: further delineation of the poison sequences Nucleic Acids Res 1986 14 8231 8245 10.1093/nar/14.21.8231 3024101 PMC311856

[B31] Zabransky A Sakalian M Pichova I Localization of self-interacting domains within betaretrovirus Gag polyproteins Virology 2005 332 659 666 10.1016/j.virol.2004.12.007 15680431

[B32] Grote A Hiller K Scheer M Munch R Nortemann B Hempel DC Jahn D JCat: a novel tool to adapt codon usage of a target gene to its potential expression host Nucleic Acids Res 2005 33 W526 531 10.1093/nar/gki376 15980527 PMC1160137

[B33] Swanson CM Puffer BA Ahmad KM Doms RW Malim MH Retroviral mRNA nuclear export elements regulate protein function and virion assembly EMBO J 2004 23 2632 2640 10.1038/sj.emboj.7600270 15201866 PMC449780

[B34] Bhadra S Lozano MM Payne SM Dudley JP Endogenous MMTV proviruses induce susceptibility to both viral and bacterial pathogens PLoS Pathog 2006 2 e128 10.1371/journal.ppat.0020128 17140288 PMC1665650

[B35] Indik S Gunzburg WH Salmons B Rouault F Mouse mammary tumor virus infects human cells Cancer Res 2005 65 6651 6659 10.1158/0008-5472.CAN-04-2609 16061645

[B36] Okeoma CM Low A Bailis W Fan HY Peterlin BM Ross SR Induction of APOBEC3 in vivo causes increased restriction of retrovirus infection J Virol 2009 83 3486 3495 10.1128/JVI.02347-08 19153238 PMC2663286

[B37] Vaidya AB Taraschi NE Tancin SL Long CA Regulation of endogenous murine mammary tumor virus expression in C57BL mouse lactating mammary glands: transcription of functional mRNA with a block at the translational level J Virol 1983 46 818 828 6304344 10.1128/jvi.46.3.818-828.1983 PMC256558

[B38] Klein KC Reed JC Lingappa JR Intracellular destinies: degradation, targeting, assembly, and endocytosis of HIV Gag AIDS Rev 2007 9 150 161 17982940

[B39] Schwartz S Campbell M Nasioulas G Harrison J Felber BK Pavlakis GN Mutational inactivation of an inhibitory sequence in human immunodeficiency virus type 1 results in Rev-independent gag expression J Virol 1992 66 7176 7182 1433510 10.1128/jvi.66.12.7176-7182.1992 PMC240411

[B40] Groom HC Anderson EC Lever AM Rev: beyond nuclear export J Gen Virol 2009 90 1303 1318 10.1099/vir.0.011460-0 19321757

[B41] Deroo BJ Archer TK Glucocorticoid receptor-mediated chromatin remodeling in vivo Oncogene 2001 20 3039 3046 10.1038/sj.onc.1204328 11420719

[B42] Bolinger C Boris-Lawrie K Mechanisms employed by retroviruses to exploit host factors for translational control of a complicated proteome Retrovirology 2009 6 8 19166625 10.1186/1742-4690-6-8 PMC2657110

[B43] Simpson SB Zhang L Craven RC Stoltzfus CM Rous sarcoma virus direct repeat cis elements exert effects at several points in the virus life cycle J Virol 1997 71 9150 9156 9371572 10.1128/jvi.71.12.9150-9156.1997 PMC230216

[B44] Simpson SB Guo W Winistorfer SC Craven RC Stoltzfus CM The upstream, direct repeat sequence of Prague A Rous sarcoma virus is deficient in mediating efficient Gag assembly and particle release Virology 1998 247 86 96 10.1006/viro.1998.9233 9683574

[B45] Ogert RA Lee LH Beemon KL Avian retroviral RNA element promotes unspliced RNA accumulation in the cytoplasm J Virol 1996 70 3834 3843 8648719 10.1128/jvi.70.6.3834-3843.1996 PMC190260

[B46] Berberich SL Macias M Zhang L Turek LP Stoltzfus CM Comparison of Rous sarcoma virus RNA processing in chicken and mouse fibroblasts: evidence for double-spliced RNA in nonpermissive mouse cells J Virol 1990 64 4313 4320 2166819 10.1128/jvi.64.9.4313-4320.1990 PMC247898

[B47] Vogt VM Bruckenstein DA Bell AP Avian sarcoma virus gag precursor polypeptide is not processed in mammalian cells J Virol 1982 44 725 730 6183452 10.1128/jvi.44.2.725-730.1982 PMC256320

[B48] Nasioulas G Hughes SH Felber BK Whitcomb JM Production of avian leukosis virus particles in mammalian cells can be mediated by the interaction of the human immunodeficiency virus protein Rev and the Rev-responsive element Proc Natl Acad Sci USA 1995 92 11940 11944 10.1073/pnas.92.25.11940 8524879 PMC40519

[B49] Swanson CM Sherer NM Malim MH SRp40 and SRp55 promote the translation of unspliced human immunodeficiency virus type 1 RNA J Virol 84 6748 6759 10.1128/JVI.02526-09 PMC2903291 20427542

[B50] Pollard VW Malim MH The HIV-1 Rev protein Annu Rev Microbiol 1998 52 491 532 10.1146/annurev.micro.52.1.491 9891806

[B51] Zolotukhin AS Michalowski D Bear J Smulevitch SV Traish AM Peng R Patton J Shatsky IN Felber BK PSF acts through the human immunodeficiency virus type 1 mRNA instability elements to regulate virus expression Mol Cell Biol 2003 23 6618 6630 10.1128/MCB.23.18.6618-6630.2003 12944487 PMC193712

[B52] Mertz JA Lozano MM Dudley JP Rev and Rex proteins of human complex retroviruses function with the MMTV Rem-responsive element Retrovirology 2009 6 10 10.1186/1742-4690-6-10 19192308 PMC2661877

[B53] Lu XB Heimer J Rekosh D Hammarskjold ML U1 small nuclear RNA plays a direct role in the formation of a rev-regulated human immunodeficiency virus env mRNA that remains unspliced Proc Natl Acad Sci USA 1990 87 7598 7602 10.1073/pnas.87.19.7598 2217190 PMC54795

[B54] Cochrane AW McNally MT Mouland AJ The retrovirus RNA trafficking granule: from birth to maturity Retrovirology 2006 3 18 10.1186/1742-4690-3-18 16545126 PMC1475878

[B55] Swanson CM Malim MH Retrovirus RNA trafficking: from chromatin to invasive genomes Traffic 2006 7 1440 1450 10.1111/j.1600-0854.2006.00488.x 16984406

[B56] Suhasini M Reddy TR Cellular proteins and HIV-1 Rev function Curr HIV Res 2009 7 91 100 10.2174/157016209787048474 19149558

[B57] Zabransky A Hadravova R Stokrova J Sakalian M Pichova I Premature processing of mouse mammary tumor virus Gag polyprotein impairs intracellular capsid assembly Virology 2009 384 33 37 10.1016/j.virol.2008.10.038 19046754

[B58] Fasel N Buetti E Firzlaff J Pearson K Diggelmann H Nucleotide sequence of the 5' noncoding region and part of the gag gene of mouse mammary tumor virus; identification of the 5' splicing site for subgenomic mRNAs Nucleic Acids Res 1983 11 6943 6955 10.1093/nar/11.20.6943 6314267 PMC326430

[B59] Fasel N Pearson K Buetti E Diggelmann H The region of mouse mammary tumor virus DNA containing the long terminal repeat includes a long coding sequence and signals for hormonally regulated transcription EMBO J 1982 1 3 7 6325151 10.1002/j.1460-2075.1982.tb01115.x PMC552986

[B60] Redmond SM Dickson C Sequence and expression of the mouse mammary tumour virus env gene EMBO J 1983 2 125 131 11894899 10.1002/j.1460-2075.1983.tb01393.x PMC555099

[B61] Xu L Sakalian M Shen Z Loss G Neuberger J Mason A Cloning the human betaretrovirus proviral genome from patients with primary biliary cirrhosis Hepatology 2004 39 151 156 10.1002/hep.20024 14752833

[B62] Sakalian M Rapp ND Rescue of internal scaffold-deleted Mason-Pfizer monkey virus particle production by plasma membrane targeting Virology 2006 345 317 327 10.1016/j.virol.2005.09.066 16297423

