# Supplementary figures and images for: Translation of MMTV Gag requires nuclear events involving splicing motifs in addition to the viral Rem protein and RmRE
Source: Retrovirology. 2012 Jan 25;9:8. doi: 10.1186/1742-4690-9-8 (PMC3292498; doi:10.1186/1742-4690-9-8)

## Slide 1
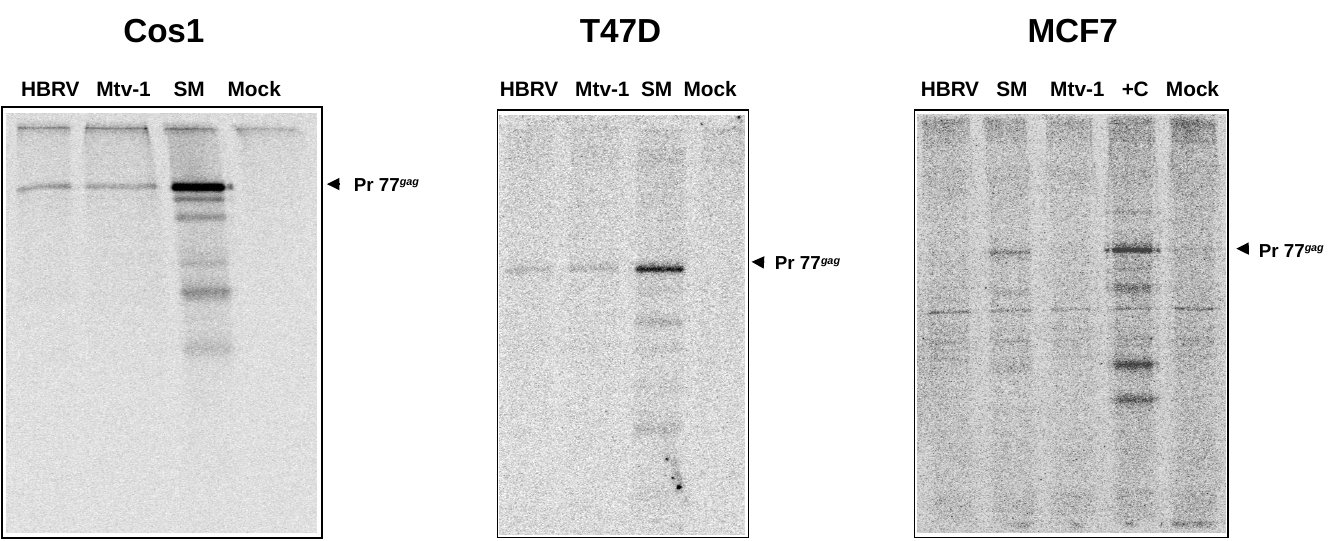

Cos1
T47D
MCF7
HBRV Mtv-1 SM Mock
HBRV Mtv-1 SM Mock
HBRV SM Mtv-1 +C Mock
Pr 77gag
Pr 77gag
Pr 77gag

Supplement: Additional file 1 — (PPT) Similar Gag expression profiles are seen in several cell types. The indicated gag constructs were transfected into COS-1 cells, and the human breast carcinoma cell lines, T47D and MCF7. After 24 hr Gag expression was assayed by metabolic labeling followed by immunoprecipitating with anti-MMTV CA antibody. +C, pSMt-HYB. Pr77gag, the Gag precursor (77 KDa) is indicated by the arrow. [file 1742-4690-9-8-S1.PPT]

## Slide 1
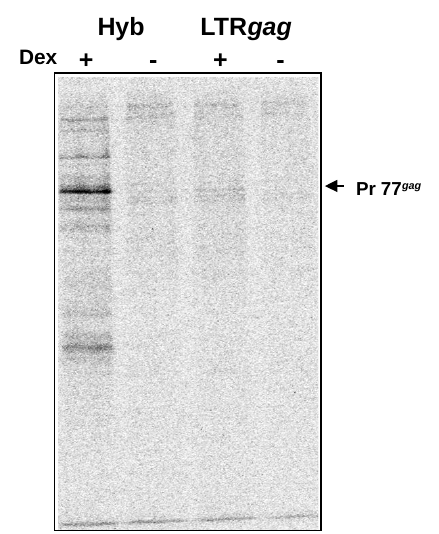

Hyb LTRgag
Dex
+ - + -
Pr 77gag

Supplement: Additional file 2 — (PPT) The cognate 5' UTR does not rescue Gag expression. MMTV Gag was expressed in HEK 293T cells either from an intact provirus (pHyb-Mtv) or from the MMTV pLTR-gag construct in the presence or absence of dexamethasone (Dex). After 24 hrs, dexamethasone was added to the cells and 48 hrs post transfection, cells were radiolabeled and Gag expression was assayed by immunoprecipitating with anti-MMTV CA antibody. Pr77gag, the Gag precursor (77 KDa). [file 1742-4690-9-8-S2.PPT]

## Slide 1
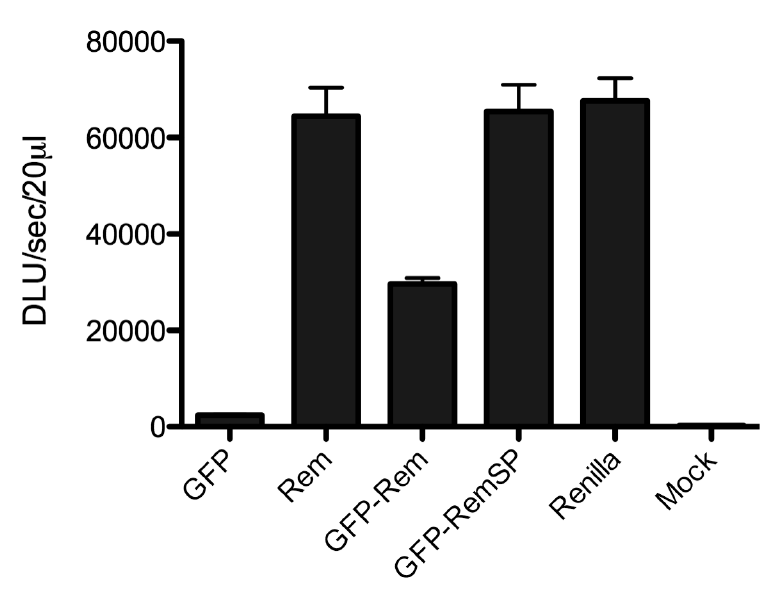

Supplement: Additional file 3 — (PPT) Rem enhances expression of a reporter gene. To verify Rem function, we used the reporter plasmid pHMRluc in which Renilla luciferase is in an intron, upstream of the Rem response element. pHMRluc was cotransfected with the indicated plasmids and luciferase activity was integrated over one second. The average of triplicate luciferase readings of three independent transfections ± SD is shown. A plasmid expressing Renilla luciferase from a CMV promoter was used as a positive control (Renilla) and mock-transfected cells were used as a negative control. The GFP lane shows the basal luciferase expression from the pHMRluc plasmid. [file 1742-4690-9-8-S3.PPT]
