# Supplementary material for: Translation of MMTV Gag requires nuclear events involving splicing motifs in addition to the viral Rem protein and RmRE
Source: Retrovirology. 2012 Jan 25;9:8. doi: 10.1186/1742-4690-9-8 (PMC3292498; doi:10.1186/1742-4690-9-8)
Supplement: Additional file 4 — (PPT) Location of RmRE elements in constructs. The viral genomic RNA from nt 6709-8564 in Mtv-1 (see Methods for accession #) is shown on the top line with the splice acceptor 2 (SA2) indicated by the orange triangle and the initiation and termination codons of Sag and Env. The two published RmRE sequences are depicted relative to their position versus the reference genome. Following the published RmRE sequences are those utilize in the current manuscript beginning with the sequence that was introduced into the CMV promoter Gag-only constructs (CRmRE) and the same construct containing splice donor and acceptor sequences (CssRmRE). The final two sequences are those that were introduced into proviral derived Gag expression constructs that lacked the splice acceptor (LTRgag) or contained a functional splice acceptor (LTR+SA). For clarity, promoter and gag sequences 5' to the area introduced to contain the RmRE and splice sites are not shown. [file 1742-4690-9-8-S4.PPT]

## Slide 1
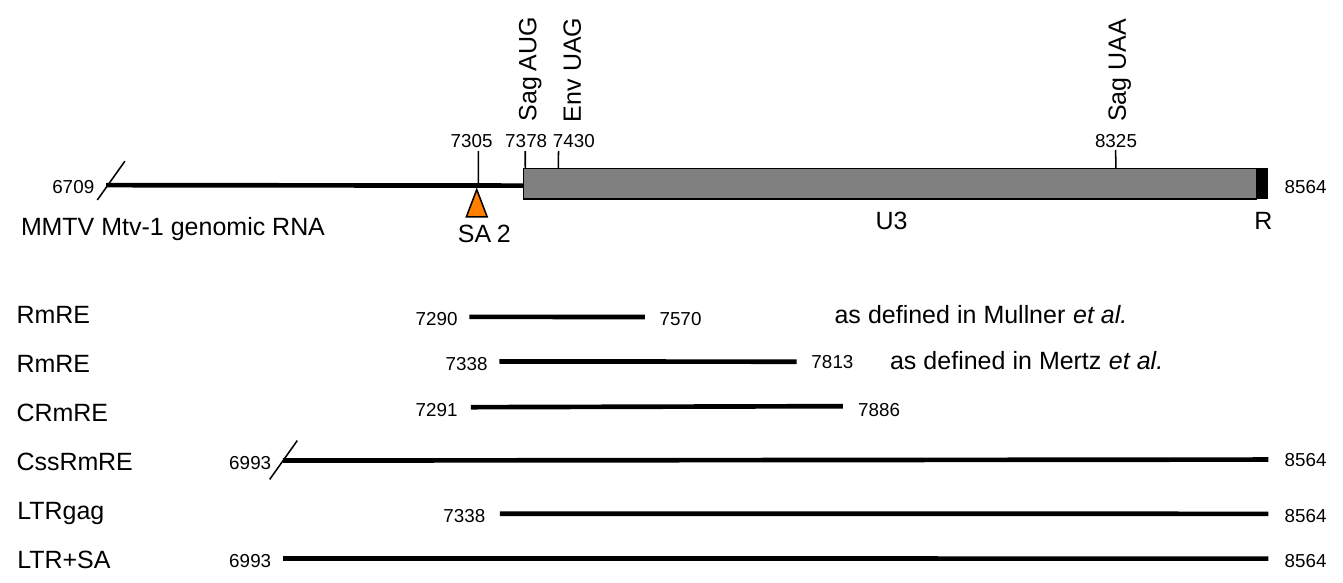

Sag AUG
Env UAG
Sag UAA
7378
8325
7305
7430
8564
6709
R
U3
MMTV Mtv-1 genomic RNA
SA 2
RmRE
as defined in Mullner et al.
7290
7570
as defined in Mertz et al.
RmRE
7813
7338
CRmRE
7291
7886
CssRmRE
8564
6993
LTRgag
7338
8564
LTR+SA
6993
8564
